# Perturbations in Dairy Cows: Impact of Heat Stress, Lameness, and Mastitis on Milk Yield and Feeding Behavior

**DOI:** 10.3390/ani16071111

**Published:** 2026-04-04

**Authors:** Anita Cabbia, Matteo Braidot, Eleonora Florit, Mirco Corazzin, Alberto Romanzin

**Affiliations:** Department of Agricultural, Food, Environmental and Animal Sciences, University of Udine, Via Delle Scienze 206, 33100 Udine, Italy; matteo.braidot@uniud.it (M.B.); florit.eleonora@spes.uniud.it (E.F.); mirco.corazzin@uniud.it (M.C.); alberto.romanzin@uniud.it (A.R.)

**Keywords:** perturbation, smoothing, milk yield, eating time, rumination time

## Abstract

Dairy cows typically respond to stressors by altering their behavior, such as reducing eating time (ET) and rumination time (RT). Although declines in milk yield (MY) have been extensively studied, models to quantify perturbations in ET and RT are still lacking. This study adopts a smoothing approach to identify and characterize perturbations in MY, ET, and RT in response to the main primary stressors, heat stress (HS), lameness (L), and mastitis (M), while evaluating the influences of parity and stage of lactation. A total of 350 Italian Simmental cows were monitored in farms equipped with automatic milking systems and accelerometers. Within this population, cows with a lactation period of at least 150 days were selected. A double-curve smoothing model (λ = 100 and λ = 10,000) was applied to calculate response and recovery times and to quantify production and feeding behavior losses. The results indicate that L causes the longest (30.6 d and 28.8 d, respectively) perturbations for both MY and ET. While L caused the greatest loss in milk production (14.7 kg), HS resulted in the greatest losses regarding feeding behavior (ET: 175.2 min and RT: 210.3 min). In general, M had a lower impact, likely due to the timeliness of treatments. Primiparous cows showed faster responses to stress but slower recovery times compared to multiparous ones. However, multiparous cows exhibited greater total MY losses. The method proved effective for quantifying resilience and opens new perspectives in health monitoring, allowing for the identification of both economic loss and each animal’s capacity to cope with pathological and environmental events, improving the overall sustainability of the dairy farm.

## Simple Summary

This study used a smoothing technique to analyze perturbations in the milk production and feeding behavior of dairy cows caused by main stressors (heat stress, lameness, and mastitis). The results indicate that lameness causes the longest and most difficult to recover from perturbations, while heat stress dramatically reduces eating and rumination times. In addition, primiparous cows show greater sensitivity and slower recovery times. These findings provide farmers with important tools to identify stressors early by improving production efficiency and welfare.

## 1. Introduction

Maintaining an efficient dairy livestock system is crucial given the increasing demand for animal-derived foods. Precision Livestock Farming relies on high-frequency data collected automatically by sensors to enhance overall production efficiency. In recent years, the widespread adoption of technology in commercial farms has integrated various devices (such as automatic milking systems, automatic feeding systems, collars, ear tags, reticulorumen boluses, and pedometers) into several production stages [1,2]. These technologies facilitate the continuous monitoring of individual productivity and health status, optimizing productive potential while reducing operational costs [3].

In the livestock sector, animal welfare and feed efficiency are now considered key factors in precision farming. Within this framework, feeding behavior is among the most monitored traits, as it is closely linked to production and health. Accelerometers, often integrated into ear tags and collars, are among the most utilized sensors for feeding behavior monitoring [4]. These devices detect feeding behavior and other activity patterns in real-time over extended periods, providing significant insights into performance, health status, and welfare [5,6,7].

Feeding behavior occupies a significant portion of the dairy cow’s daily budget and is primarily divided into eating time (ET) and rumination time (RT) [8,9]. ET and RT are widely used in dairy farming as indicators of animal health [10,11]. Studies finding a direct relationship with production performance are less common, but positive relationships have been identified between both ET and RT with milk yield (MY) [12,13].

Cows respond to stressors through complex behavioral changes, such as the reduction in ET and RT, variation in resting time, or modification in locomotion activity, with the specific response direction depending on the nature of the stressor [7]. Heat stress (HS) is a topic of particular focus, especially in relation to global warming. HS compromises MY [14], welfare [15], and feed intake [16]. However, the relationship between HS and feed efficiency remains debated: while some studies report reduced efficiency in heat-stressed dry cows [17], others found no significant association [18].

A second stressor for dairy cows is lameness (L), a prevalent disease among cows and considered a cause of chronic stress [19]. It causes pain and behavioral changes in terms of abnormal locomotion, leading to a decrease in ET [20]. A recent study [21] reports that L is a major driver of economic loss, causing a reduction in MY of up to 20%.

Mastitis (M) remains one of the most prevalent and costly pathologies [22]. Beyond clinical indicators like increased somatic cell count (SCC), it significantly influences cattle behavior [23,24]. In general, affected cows exhibit restlessness during milking and reduce recumbency time [25]. Furthermore, several studies have shown that cows with M modify their feeding behavior by reducing ET and RT [26,27,28].

All these stressors reduce animal welfare and profit for dairy farmers. Given that ET and RT are intrinsic to health and performance, analyzing these traits is vital during pathological events. Consequently, there is a compelling need to investigate how individual animals respond to different stressors. Previous research has already assessed their corresponding response and recovery on MY. Codrea et al. [29] proposed a smoothing approach to extract biologically significant features from MY data. This method can provide the means to examine hypotheses concerning the nature of deviations, as it can detect and quantify the alteration in MY. In addition, Ben Abdelkrim et al. [30] extended this by using a differential smoothing approach to estimate unperturbed performance trajectories and subsequently identify the phenotypic features of perturbations. However, to our knowledge, there are no studies applying this method to ET and RT.

The aim of this study was to quantify the impact of prevalent stressors related to MY and subsequently adapt the same analytical framework to ET and RT data, taking into account parity and the stage of lactation.

## 2. Materials and Methods

### 2.1. Animals and Data Collection

The study monitored five commercial dairy farms in northeastern Italy (Friuli Venezia Giulia region) over a 12-month period, involving a total of 350 Italian Simmental dairy cows. The following inclusion criteria were applied for farm selection: all dairy cows were housed in a free-stall system equipped with a solid floor, alley scraper, and high-volume low-speed fans and were milked via an automatic milking system (AMS). Furthermore, each cow was equipped with a specialized sensor to monitor feeding behavior (SenseHub Dairy, Allflex Livestock Intelligence, Merck & Co., Rahway, NJ, USA).

Within this population, only cows with a lactation period of at least 150 days (from days in milk (DIM) = 0 to DIM ≥ 150) and for which complete daily MY, ET, and RT data were available were selected. This resulted in 182 cows being considered for the study of MY; among these, 70 were primiparous and 112 were multiparous cows. Regarding ET, 157 cows were included, of which 53 were primiparous and 104 were multiparous. For RT, 133 cows were considered, including 48 primiparous and 85 multiparous. The selected animals had the following characteristics: parity (number of lactation): 2.4 ± 1.5; DIM: 129 ± 81 days; MY: 28.9 ± 8.1 kg/d; fat content: 4.0 ± 0.7%; and protein content: 3.6 ± 0.3%. Lactation was divided into three stages: the first was from 0 to 100 days, the second from 101 to 200, and the third from 201 to 305 [31].

Raw feeding behavior data was continuously recorded in 2 h blocks, uploaded to a processing unit, and aggregated into daily values (minutes/day). Performance data such as DIM, parity, MY, milk composition (fat, protein, and lactose), and SCC, alongside feeding behavior parameters (ET and RT), were extracted from the AMS management software (Horizon v. 4.12.5.609, Lely Industries, Maassluis, The Netherlands).

### 2.2. Stressors Definition

HS was determined via the Temperature Humidity Index (THI), calculated from ambient temperature and humidity data recorded by dedicated devices, following NRC [32]:

$$THI=\left[\left(1.8*T\right)+32\right]-[\left(0.55-(0.0055*RH\right))*\left(1.8*T-26\right)]$$

where T is the environmental temperature in °C and % RH is the relative humidity.

Following Collier et al. [33], THI levels were categorized as no HS (≤72) and HS (≥72). Notably, the THI did not exceed 80 during the study. L was detected using locomotion scores (LSs), as proposed by Sprecher et al. [34], distinguishing between normal locomotion (1 ≤ LS ≤ 3) and severe L (4 ≤ LS ≤ 5). Lame cows were assumed to be in that state for a 14-day window centered on the assessment date (L ± 7 days) [35]. Finally, M was identified using thresholds of SCC [36]: healthy (<100,000 cells/mL) and infected (≥100,000 cells/mL) cows.

### 2.3. Data Analysis

The analysis aimed to identify, quantify, and characterize perturbations in MY, ET, and RT, defined as events involving a loss of MY or duration (ET and RT). To ensure a consistent dataset, input data were pre-processed by filtering individual lactations. If ET and RT values were missing, the time series was truncated at the first persistent missing data point to ensure continuity. For each variable and animal, two trend curves were generated using a smoothing approach with two different smoothing parameters (λ): λ = 10,000 to represent the overall long-term trend and λ = 100 to capture short-term variations. The function to be minimized is the one reported by Codrea et al. [29], and the λ are those suggested by the same author. The first and second derivatives were calculated based on this curve.

**Figure 1:**
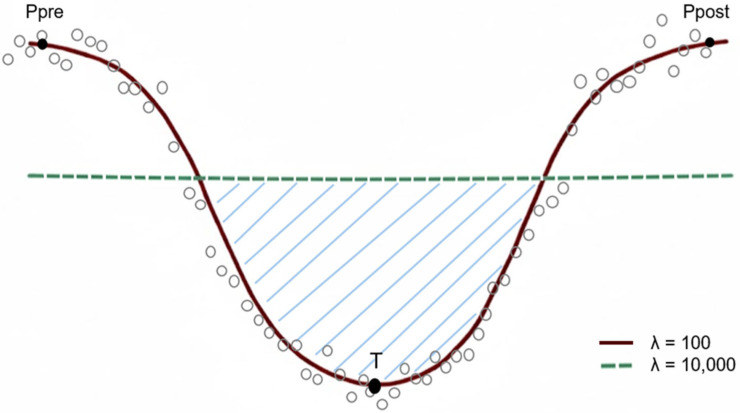
Example of the curve points considered for defining the onset (Ppre), offset (Ppost), and trough (T) of a perturbation. Response represents the period from Ppre to T, while recovery is the time from T to Ppost. The grey circles represent the daily records. The loss was quantified as the area (blue lines) between the dashed and solid curves during the perturbation interval.

A perturbation event was defined when the λ = 100 curve dropped below the λ = 10,000 curve, ending when the first one crossed back above the other curve. For each perturbation (Figure 1), the inflection points (sign changes in the first derivative) were used to characterize the dynamics of response and recovery.

- Ppre: DIM, where the trend shifts from positive to negative preceding the event.
- T: DIM of the minimal value (change from negative to positive) within the event.
- Ppost: DIM, where the trend shifts from positive to negative following the event.

Response represents the period from Ppre to T, while recovery is the time from T to Ppost. The loss (such as MY, ET, and RT) was quantified as the area between the two curves during the perturbation interval. Finally, perturbations were attributed to specific stressors (HS, L, and M). Events that do not correspond to any specific cause were marked as NA (not assigned). In cases of concurrent stressors, the prevalent one was determined by the day of maximal loss (corresponding to the peak of the event). Perturbations whose cause emerged more than three days after the onset of the event were also classified as NA.

### 2.4. Statistical Analysis

The data were analyzed in R version 4.5.1 [37]. The initial model considered a linear mixed-effect model, with the fixed effects of stage of lactation (early, mid, and late), parity (primiparous, multiparous), and stressor (HS, L, M, and NA); also, the interactions between stage of lactation × stressor and parity × stressor were included. Furthermore, the random effect of animals nested within farms was included. Normality of residuals was assessed with the Shapiro–Wilk test. Homogeneity of variance was evaluated using simulation-based residual diagnostics, as implemented in the DHARMa package (v. 0.4.7) [38]. When these assumptions were violated, transformations (logarithmic and Box–Cox) were applied. If the assumptions were still not met, the same model, but with a generalized approach, was used. Simplified, more parsimonious models (without interactions) were also tested, and model selection was guided by AIC. The final model included the fixed effect of stage of lactation, parity, and stressor and the random effect of animals nested within the farm. The only exceptions were the recovery speed (MY) and the relative loss (MY), where the final model also included the parity × stressor interaction. *p*-values for multiple comparisons were adjusted using the false discovery rate (FDR) method.

## 3. Results

### 3.1. Duration of Perturbations

Table 1 reports the total duration of perturbations, the time to response to the cause of the perturbation, and the recovery time, studied across three variables: MY, ET, and RT.

Regarding total duration, parity did not significantly influence any of these three variables. Conversely, the total duration of production perturbations in the MY variable was statistically significant as a function of the stage of lactation (*p* < 0.01). Perturbations occurring during the first stage of lactation were shorter (26.0 d) compared to those in the second and third stages (28.9 d and 28.2 d, respectively). The total duration of perturbations based on the triggering stressors was significant for all of the variables considered. L was identified as the stressor leading to the longest perturbation for MY (30.6 d; *p* = 0.01). For the ET, HS was found to be a high-impact stressor, along with L. Specifically, L resulted in perturbations of 28.8 d (*p* < 0.01) on ET, while HS resulted in perturbations of 26.6 d (*p* < 0.01).

The time taken for the variables to respond to the perturbation and the subsequent time for recovery also showed significant differences across factors. Response times in MY and ET were affected by parity and the stage of lactation. The response time was longer in multiparous cows compared to primiparous cows both for MY (17.1 d and 14.8 d, respectively; *p* < 0.01) and ET (12.7 d and 11.9 d, respectively; *p* = 0.04). MY showed longer response times in the second (16.8 d) and the third stages (17.8 d) compared to the first (13.4 d; *p* < 0.01). ET also showed the longest response time in the third stage (13.9 d), which was different from the first stage (10.5 d; *p* < 0.01). The response time showed significant effects across stressors for MY and RT but not for ET (*p* = 0.02, *p* < 0.01, and *p* > 0.11, respectively). Regarding MY, HS (17.2 d) resulted in the longest response time, being longer than M (15.0 d) and NA (15.3 d) stressors. Similarly, for RT, HS (12.3 d) resulted in a longer response time than NA (10.3 d).

Recovery time was significantly influenced by all three factors, parity, stage of lactation, and stressors, but only for MY and ET. For both parameters, primiparous cows exhibited a longer recovery time (MY: 12.2 d, *p* < 0.01; ET: 14.0 d, *p* = 0.03) compared to multiparous (MY: 10.7 d; ET: 13.0 d). For MY, the longest recovery time was observed in the first (12.3 d) and in the second stage (11.8 d), which were greater compared to the third stage (10.2 d; *p* < 0.01). ET followed a similar trend, where the first stage (15.8 d) demonstrated a longer recovery time compared to the second (12.7 d) and the third stage (12.2 d; *p* < 0.01). Finally, the analysis of stressors highlighted that L resulted in the longest recovery time for both MY (14.2 d) and ET (15.5 d). Conversely, HS was associated with the shortest recovery time for MY (10.1 d; *p* < 0.01), and M showed a shorter recovery time for ET (12.2 d; *p* = 0.01) compared to L. Considering the recovery speed for MY, the parity × stressor interaction was significant (*p* = 0.02); in particular, in multiparous cows, the recovery speeds of L and M were higher than that of NA (0.13 ± 0.013 kg/d vs. 0.10 ± 0.005 kg/d; *p* < 0.01 and 0.14 ± 0.007 kg/d vs. 0.10 ± 0.005 kg/d; and *p* < 0.01, respectively), and the recovery speed of M was superior to that of HS (0.14 ± 0.007 kg/d vs. 0.10 ± 0.008 kg/d; *p* < 0.01). Furthermore, the highest value was observed in the first stage of lactation (*p* < 0.05). Recovery speed for ET was higher in primiparous cows (*p* < 0.01), in the first stage of lactation (*p* < 0.01), and in HS compared to other stressors (*p* < 0.05).

### 3.2. Milk Yield Loss and Change in Feeding Behavior

The effects of parity, stage of lactation, and stressors on mean MY, ET, and RT losses, including their respective loss intensities, are presented in Table 2. Parity significantly influenced MY, with multiparous cows exhibiting a higher mean MY (12.1 kg) compared to primiparous cows (10.3 kg; *p* < 0.01). With respect to the stage of lactation, the quantity of milk lost due to the perturbation was higher in the first stage (13.3 kg) compared to both the second (10.3 kg) and third stages (10.1 kg; *p* < 0.01). Regarding the stressors, perturbations caused by L resulted in the highest MY loss (14.7 kg), which was higher than NA (9.2 kg; *p* < 0.01).

Loss ET was influenced by parity, being longer for primiparous cows (154.1 min) than for multiparous cows (130.5 min; *p* < 0.01). No statistically significant differences in this variable were observed when considering the stage of lactation. The analysis of the stressors indicated that HS registered the greatest loss in ET (175.2 min), a value higher than that of cows affected by M (129.9 min) and NA (120.5 min; *p* < 0.01).

Neither parity nor the stage of lactation was found to be statistically significant regarding the loss of RT. However, the stressors showed statistical significance for the different causes considered (*p* < 0.01). Mean RT loss was higher in HS than in NA (210.3 min and 134.1 min, respectively; *p* < 0.01).

The intensity of MY loss was influenced by parity, being greater in multiparous cows (0.80 kg/d) compared to primiparous cows (0.70 kg/d; *p* < 0.01). When analyzing the stage of lactation, in the first stage, the loss intensity was greater (0.84 kg/d) than in the second (0.68 kg/d) and third stages (0.73 kg/d; *p* < 0.01). The stressor that resulted in the most intense MY loss was L (0.94 kg/d), and the least intense loss was observed for the NA stressor (0.62 kg/d; *p* < 0.01).

Regarding the intensity of ET minutes lost, all three factors considered were found to be significant. The loss intensity for primiparous cows was higher compared to multiparous cows (9.80 min/d vs. 8.70 min/d, respectively; *p* < 0.01). The stage of lactation showed the maximum intensity in the first stage (9.60 min/d) and differed from the second stage (8.80 min/d; *p* = 0.03). Concerning the cause of perturbation, the loss intensity of ET was maximal in cows affected with HS (10.80 min/d), differing from the other stressors (*p* < 0.05).

The loss intensity of RT showed no significant differences between parities or between stages of lactation. However, two stressors had a significant impact on the intensity of minutes lost, L (16.40 min/d) and HS (14.90 min/d), while the lowest values were recorded for cows with M (11.60 min/d) and NA (10.30 min/d; *p* < 0.01).

Considering the relative loss of MY, the interaction parity × stressor was significant (*p* = 0.04); in particular, in primiparous cows, the MY relative loss related to L was higher than that related to M (5.72 ± 1.990% vs. 2.50 ± 0.248%; *p* = 0.04), NA (5.72 ± 1.990% vs. 2.28 ± 0.165%; *p* = 0.04), and HS (5.72 ± 1.990% vs. 2.42 ± 0.226%; *p* = 0.04). Furthermore, the MY relative loss was highest in the third stage of lactation (*p* < 0.01).

## 4. Discussion

### 4.1. Smoothing Approach

The present study applied an analytical framework based on smoothing techniques to quantify the influence of three common stressors (HS, L, and M) on MY and on feeding behavior parameters (ET and RT) in dairy cows. The objective was to characterize not only the magnitude of MY loss (kg) or ET and RT loss (minutes) but also the temporal dynamics of response and recovery from these stressors. Furthermore, we investigated how the effect of these stressors changes according to parity and the stage of lactation. The results clearly indicate that the nature of the stressor generates a differential impact on the animal. The most innovative aspect of this work is the perturbation detection starting from daily MY, ET, and RT data, as few scientific studies currently identify methods to detect and quantify perturbations. Among these, Codrea et al. [29] defined a system based on a smoothing approach to detect deviations in MY and to characterize and quantify these fluctuations by inducing stress through dietary changes. More recently, Ben Abdelkrim et al. [30] developed a perturbed lactation model to quantify animal resilience by analyzing deviations from the potential lactation curve. Regarding MY in our study, we built upon these frameworks, implementing adaptations necessary for application to the whole lactation period. To our knowledge, no studies have developed analogous systems for ET and RT. Therefore, the same system was applied to these parameters without further variations. Currently, the main limitation of this tool concerns the data collection timeframe, as processing via RStudio (v. 4.5.1) and assigning perturbation causes are time-consuming. However, new AI-based approaches may facilitate the practical application of this system. Furthermore, in some cases, we observed that the model did not correctly capture perturbations in late lactation. Consequently, the tool is not always able to adequately distinguish a loss of milk production from the physiological drop that occurs in late lactation. To overcome this limitation, dynamic lambda values could be used with more strict values as the DIM increases, reducing the effect of the drying-off process on the baseline. However, the application of such dynamic models will require specific studies.

### 4.2. Parity and Stage of Lactation Effect on Perturbations

The characteristics of MY and ET perturbations varied significantly as a function of stage of lactation and parity, aligning with the findings of Adriaens et al. [39] and Wang et al. [40]. Although the overall duration of the perturbation was not statistically different between primiparous and multiparous cows, the analysis of response and recovery times shows crucial differences. This suggests that, while the total time the stressor exerts its effect is similar, the time required to return to normality differs based on the cattle’s individual capacity. Our results indicate that perturbations in the early stage of lactation and in first parity generally have a faster response (the negative effect manifests sooner) but demonstrate a slower recovery. The recovery speed for MY was different across the three stages of lactation, further clarifying these dynamics. The higher recovery speed observed in the first stage of lactation suggests an intense compensatory mechanism to offset losses; however, the absolute recovery time remains long. This phenomenon can be partially explained by the increased susceptibility to clinical pathologies and the critical nature characterizing early lactation and first parity. Primiparous cows are still growing, and their immune systems are more immature than those of multiparous cows [41]. Furthermore, early lactation cows are more exposed to a negative energy balance, which compromises their recovery ability and makes them more likely to develop L due to physiological and management changes [39,42,43].

MY losses were higher for animals with a higher number of lactations, consistent with Carvalho et al. [44] and Adriaens et al. [39], likely because multiparous cows produce more milk [45], resulting in a greater absolute MY loss, but the relative loss did not differ between primiparous and multiparous cows. For the same reason, the greatest MY loss occurred in the first stage of lactation when milk production was higher, but in terms of relative loss, it prevails in late lactation. The greater ET loss in primiparous cows may result from the greater difficulty in resuming normal feed intake (in terms of digestive volume) in the postpartum period combined with higher ET values achieved throughout lactation [46]. Furthermore, Jaeger et al. [47] observed a 4.5%/d reduction in ET in multiparous cows compared to primiparous ones; consequently, multiparous cows can feed more efficiently, achieving a higher DMI in less time.

### 4.3. Stressor Effect on Perturbations

L emerged as the most critical stressor for MY, with a significantly longer total duration compared to the other causes examined. This persistence is linked to the painful and often chronic nature of L, which negatively affects behavior, consistent with several studies [20,48,49]. The impact on movement reduces the cow’s propensity to access AMS [50], leading to decreased AMS visits [51] and, consequently, reduced MY and concentrate intake [52]. Spörndly and Wredle [53] suggest that L may be one of the most important factors influencing the frequency of cow visits to AMSs. This severity is also reflected in the intensity of the productive loss: Warnick et al. [54] reported that MY drops from 0.8 to 1.5 kg/d in the case of L. Interestingly, L affects MY differently, depending on the parity. In particular, primiparous cows were more influenced, with greater MY losses, even in relative terms. Multiparous cows, however, showed a faster recovery speed.

Regarding the consequences on feeding behavior, our data show that minutes of ET and RT decreased in the presence of L. It is crucial to note, however, that the impact on duration was much more persistent for ET than for RT. This suggests that the pain associated with L penalizes standing behaviors (such as eating) more severely, as the cow seeks to minimize limb load. This is consistent with the study by Lemmens et al. [55], which reported a significant decrease. The higher vulnerability of ET confirms the importance of the rapid treatment of L to maintain DMI. Conversely, our finding of a decrease in RT is consistent with Džermeikaitè et al. [56], indicating that, although rumination can be performed while recumbent, it is still negatively influenced by the general state of disease.

The chronic impact of L is further confirmed by recovery dynamics, where this cause determines the longest recovery times for both the MY variable and the ET variable (but not for RT) compared with all other stressors analyzed. Concerning the response time, our results show that a cow affected by HS exhibits a slower response compared to other stressors. This is likely because, in summer, heat waves often extend over several days, increasing in severity over time, and the cumulative effect of environmental stress requires more time to fully manifest in production. However, it is important to note that, unlike L, HS is associated with a shorter recovery time for MY. This suggests that, once the environmental stressor is resolved, recovery is rapid, as there is no chronic tissue damage delaying healing, which is typical of L.

The decline in MY is a well-recognized consequence of HS; this stressor also impairs feeding behavior, reproduction [57], and metabolic health, specifically regarding respiratory alkalosis, ketosis, and ruminal acidosis [58]. Corazzin et al. [59] reported that HS causes animals to reduce the time spent on eating and leads to a 15% reduction in DMI. While M represents one of the primary causes of reduced MY and induces various physiological alterations [60], in the present study, it was found to have a lesser effect compared to the other stressors. This could be explained by the early identification of M by farmers and its timely treatment. Furthermore, the lower impact on ET and RT parameters could be related to the absence of locomotor pain, which, on the contrary, is a typical sign of L.

As mentioned initially, the primary causes leading to milk loss are HS [61], L [55], and M [62], all of which were examined in this study. Indeed, other potential causes of perturbation (i.e., NA) usually had a lesser impact on both MY and feeding behaviors.

### 4.4. Study Limitation

Several aspects linked to this approach were discussed in the previous paragraphs. Together with these limitations, another element to consider involving is the use of detailed data related to pharmacological interventions. These treatments boost the recovery process, accelerating the return to baseline levels. The implantation of further data, such as clinical treatments, could increase the sensitivity and reliability of the smoothing method proposed, providing more specific and robust resilience indicators for Precision Livestock Farming applications. This study represents the first application of a smoothing technique to behavioral data from dairy cows, and further studies will be able to develop appropriate adaptations and implementation, strengthening this type of approach.

## 5. Conclusions

The analytical framework, based on smoothing techniques, proved to be an effective and innovative tool for detecting, quantifying, and characterizing perturbations. By integrating data from sensors, this approach moved beyond the simple observation of yield drops, offering detailed insights into the temporal dynamics of animal response and recovery. The application to MY as well as feeding behavior parameters highlighted how different stressors distinctly affect animal welfare and efficiency. The method revealed that L emerges as the most critical challenge in terms of chronicity and persistence, requiring the longest recovery times, underscoring the need for early intervention. While HS shows slower response dynamics but with an immediate and intense impact on feeding activity, it allows for rapid recovery once the environmental stressor is resolved. In addition, parity and the stage of lactation significantly modulate resilience, with primiparous cows displaying higher vulnerability and slower recovery during early lactation. Implementing such analytical protocols opens new perspectives for continuous health monitoring, allowing for the identification of not only the economic loss but also the intrinsic capacity of each animal to cope with pathological and environmental events.

## Figures and Tables

**Table 1:** Effect of parity, stage of lactation, and stressors (HS, L, and M) on the duration of perturbations.

	Parity		Stage of Lactation			Stressor				RSD	*p*-Value		
	1	2	1	2	3	HS	L	M	NA		Parity	Stage of Lactation	Stressor
Duration, d													
MY	27.2	28.2	26.0 ^B^	28.9 ^A^	28.2 ^A^	27.8 ^ab^	30.6 ^a^	26.4 ^bc^	26.0 ^c^	9.93	0.17	<0.01	0.01
ET	26.1	25.7	26.2	25.4	26.2	26.6 ^A^	28.8 ^A^	23.7 ^B^	24.9 ^B^	9.14	0.51	0.37	<0.01
RT	23.0	23.2	22.6	23.2	23.6	24.4	22.7	23.0	22.5	8.26	0.74	0.42	0.08
Response, d													
MY	14.8 ^B^	17.1 ^A^	13.4 ^B^	16.8 ^A^	17.8 ^A^	17.2 ^a^	16.2 ^ab^	15.0 ^b^	15.3 ^b^	8.73	<0.01	<0.01	0.02
ET	11.9 ^b^	12.7 ^a^	10.5 ^c^	12.7 ^b^	13.9 ^a^	12.7	13.2	11.3	12.0	6.53	0.04	<0.01	0.11
RT	11.0	11.2	10.7	11.3	11.5	12.3 ^A^	10.5 ^BC^	11.4 ^AB^	10.3 ^C^	5.30	0.72	0.23	<0.01
Recovery, d													
MY	12.2 ^A^	10.7 ^B^	12.3 ^A^	11.8 ^A^	10.2 ^B^	10.1 ^c^	14.2 ^a^	11.3 ^b^	10.5 ^bc^	6.08	<0.01	<0.01	<0.01
ET	14.0 ^a^	13.0 ^b^	15.8 ^A^	12.7 ^B^	12.2 ^B^	13.8 ^ab^	15.5 ^a^	12.2 ^c^	12.6 ^bc^	7.17	0.03	<0.01	0.01
RT	12.0	12.0	12.0	11.9	12.0	12.1	12.1	11.6	12.0	6.12	0.99	0.99	0.77
MY, kg/d *	0.11	0.12	0.14 ^A^	0.11 ^B^	0.11 ^B^	0.10	0.14	0.13	0.11	0.011	0.20	<0.01	0.07
ET, min/d	2.16 ^A^	1.85 ^B^	2.30 ^A^	1.92 ^B^	1.79 ^B^	2.37 ^a^	1.81 ^b^	2.01 ^b^	1.84 ^b^	2.543	<0.01	<0.01	<0.01
RT, min/d	2.95	2.96	2.85	2.86	3.14	2.92	3.52	2.81	2.55	4.760	0.73	0.24	0.06

^A, B, C^: means in the same row and within the factor with different superscripts are significantly different (*p* < 0.01); ^a, b, c^: means in the same row and within the factor with different superscripts are significantly different (*p* < 0.05). Parity: 1 = primiparous, 2 = multiparous; stage of lactation 1 = 0–100, stage of lactation 2 = 101–200, stage of lactation 3 = 201–305; HS: heat stress; L: lameness; M: mastitis; and NA: not assigned. Abbreviations: RSD: residual standard deviation; MY: milk yield; ET: eating time; RT: rumination time; and * interaction parity × stressor was significant (*p* = 0.02).

**Table 2:** Effect of parity, stage of lactation, and stressors (HS, L, and M) on milk yield, eating time, and rumination time losses.

	Parity		Stage of Lactation			Stressor				RSD	*p*-Value		
	1	2	1	2	3	HS	L	M	NA		Parity	Stage of Lactation	Stressor
Loss													
MY, kg	10.3 ^B^	12.1 ^A^	13.3 ^A^	10.3 ^B^	10.1 ^B^	10.7 ^B^	14.7 ^A^	10.8 ^B^	9.2 ^C^	10.30	<0.01	<0.01	<0.01
ET, min	154.1 ^A^	130.5 ^B^	152.8	132.6	140.7	175.2 ^A^	147.4 ^AB^	129.9 ^B^	120.5 ^B^	120.67	<0.01	0.08	<0.01
RT, min	173.2	178.2	178.7	164.2	184.9	210.3 ^A^	218.1 ^AB^	154.9 ^BC^	134.1 ^C^	150.57	0.65	0.25	<0.01
Loss intensity													
MY, kg/d	0.70 ^B^	0.80 ^A^	0.84 ^A^	0.68 ^B^	0.73 ^B^	0.70 ^B^	0.94 ^A^	0.77 ^AB^	0.62 ^C^	0.551	<0.01	<0.01	<0.01
ET, min/d	9.80 ^A^	8.73 ^B^	9.60 ^a^	8.78 ^b^	9.41 ^ab^	10.85 ^a^	8.85 ^b^	9.10 ^b^	9.27 ^b^	5.93	<0.01	0.03	<0.01
RT, min/d	12.87	13.23	13.07	12.26	13.87	14.88 ^a^	16.39 ^a^	11.57 ^b^	10.27 ^b^	8.584	0.61	0.12	<0.01
Relative Loss													
MY, % *	2.98	2.73	2.75 ^b^	2.56 ^b^	3.30 ^a^	2.52	4.54	2.73	2.13	1.981	0.40	<0.01	0.06

^A, B, C^: means in the same row and within the factor with different superscripts are significantly different (*p* < 0.01); ^a, b^: means in the same row and within the factor with different superscripts are significantly different (*p* < 0.05). Parity: 1 = primiparous, 2 = multiparous; stage of lactation 1 = 0–100, stage of lactation 2 = 101–200, stage of lactation 3 = 201–305; HS: heat stress; L: lameness; M: mastitis; and NA: not assigned. Abbreviations: RSD: residual standard deviation; MY: milk yield; ET: eating time; RT: rumination time; and * interaction parity × stressor was significant (*p* = 0.04).

## Data Availability

All data are available from the corresponding author on reasonable request.
